# Painful Small Fiber Neuropathy Associated With Teriflunomide: A Case Series and Literature Review Related to Teriflunomide and Leflunomide

**DOI:** 10.7759/cureus.45079

**Published:** 2023-09-12

**Authors:** Ahmed Elrefaey, Anza B Memon

**Affiliations:** 1 Neurology, Faculty of Medicine, Ain Shams University, Cairo, EGY; 2 Neurology, John D. Dingell VA Medical Center, Detroit, USA; 3 Neurology, Wayne State University School of Medicine, Detroit, USA; 4 Neurology, Henry Ford Health, Detroit, USA

**Keywords:** disease-modifying therapies, multiple sclerosis, peripheral neuropathy, leflunomide, teriflunomide

## Abstract

Teriflunomide and its prodrug, leflunomide, are disease-modifying medications used to treat relapsing-remitting multiple sclerosis (RRMS) and rheumatoid arthritis (RA), respectively. Peripheral neuropathy is a rare side effect associated with both medications, although the incidence rate and exact pathological mechanism remain unknown. We present a retrospective case series of three patients with RRMS, who developed painful small fiber neuropathy at various timeframes (<6 months, one year, and four years, respectively) while on teriflunomide treatment (14 mg/day); we also engage in a literature review of small and large fiber neuropathy associated with teriflunomide and leflunomide use. All three patients developed small fiber neuropathy following teriflunomide exposure. Laboratory workup was negative for metabolic, infectious, vitamin deficiency-related, and autoimmune etiologies, except for one patient who had chronic metabolic syndromes (impaired glucose, hyperlipidemia) before medication intake. However, the patient developed neuropathy following teriflunomide treatment. Electrophysiological findings were negative for large fiber neuropathy in all three patients with positive skin biopsy, with reduced epidermal nerve fiber density (ENFD) in two of the three patients. Teriflunomide was discontinued in all cases, after which symptoms stabilized. Current literature on leflunomide supports a direct neurotoxic effect or buildup of toxic intermediates from uridine synthesis inhibition. Cessation of teriflunomide use in the described cases resulted in symptom stabilization. Early recognition and treatment may lead to good clinical outcomes in these patients.

## Introduction

Peripheral neuropathy is a nervous system disorder with a broad spectrum of clinical manifestations, ranging from positive symptoms like pain, tingling, paresthesia, and dysesthesia to negative symptoms like numbness. It can be clinically categorized into large fiber peripheral neuropathy, loss of sensation, poor balance and weakness, and small fiber neuropathy, pain, tingling, burning, and cold sensation [1]. Leflunomide is a disease-modifying drug typically recommended in active cases of rheumatoid arthritis (RA) and known to cause large fiber peripheral neuropathy. Its effects on both sensory and motor nerve fibers can be recognized based on an abnormal electrophysiological study [2]. Cessation of drug use generally results in symptom stabilization or improvement, which further reiterates its role in instigating neuropathy [3].

Teriflunomide, the active metabolite of leflunomide, possesses similar immunosuppressant and anti-inflammatory potential. It was approved by the FDA in 2012 for the treatment of relapsing-remitting multiple sclerosis (RRMS) [4]. By inhibiting dihydroorotate dehydrogenase, it imposes a cytostatic effect on T and B lymphocytes, thereby mitigating their proliferation without severely compromising the immune system [5]. Both pyrimidine synthesis inhibitors, leflunomide and teriflunomide, presumably pose comparable risks for large fiber neuropathy. However, while significant data is available on leflunomide-induced peripheral neuropathy, little is known about the effects of teriflunomide on the nervous system. Incidents of peripheral polyneuropathy were found in pooled data from the clinical trial database of the disease-modifying antirheumatic drugs and the FDA-reported post-marketing data. Still, no case reports have been published so far in the literature [6,7].

In this small, single-site, retrospective observational case series, we present three painful small fiber neuropathy cases following teriflunomide treatment at various timeframes. We also provide a comprehensive review of data on small and large fiber peripheral neuropathy associated with teriflunomide and its mother drug, leflunomide.

## Case presentation

Case 1

A 61-year-old woman with an eight-year history of RRMS presented with neuropathy in her feet after four years of teriflunomide treatment. She reported a burning sensation in her legs from her calves down and tingling, sharp shooting pain in her feet. Before teriflunomide therapy, she had failed trials of disease-modifying therapies (DMTs) due to various side effects, including injection site reactions with glatiramer acetate, liver enzyme elevation with interferon beta-1a, and nausea and vomiting with dimethyl fumarate.

Neurological examination showed features of small fiber neuropathy with reduced sensation to pinprick a few centimeters below the ankles down to her toes and mildly reduced sense of vibration distally in her toes with normal position sense. Motor examination was normal. Deep tendon reflexes (DTR) were normal (2+) in the upper and lower extremities. Serum protein electrophoresis and immunofixation showed IgG lambda monoclonal protein (<0.1 g/dL). Plasma cell dyscrasias were excluded following a negative skeletal survey and a 24-hour urine protein collection, and the patient was diagnosed with monoclonal gammopathy of unknown significance (MGUS). Electromyography (EMG) and nerve conduction studies (NCS) were negative for large fiber peripheral neuropathy. A skin biopsy revealed significantly reduced epidermal nerve fiber density (ENFD) in a sample taken from the left foot, consistent with small fiber neuropathy, and normal ENFD in the left calf (Table 1). Gabapentin was initiated with a 300 mg daily dose and titrated up to 600 mg three times/day due to poor pain control. The patient could not tolerate further dose escalation. 

**Table 1:** Epidermal nerve fiber density test results for patient 1 (nerve fiber per millimeter)

Specimen	Patient value	Abnormal	Low normal
Left foot	1.5	<3	3–4.8
Left calf	5.4	<4	4–5.2

Teriflunomide therapy was discontinued, and lower extremity pain and paresthesia stabilized without progression.

Case 2

A 54-year-old woman with a three-year history of RRMS presented with neuropathy in her feet after one year of teriflunomide treatment. She reported sharp stabbing pain and tingling sensation from the calves down to the feet. Before teriflunomide therapy, she had failed trials of DMTs due to various side effects, including injection site reactions with glatiramer acetate and severe leukopenia with dimethyl fumarate. 

Neurological examination showed features of small fiber neuropathy with reduced sensation to pinprick a few centimeters below the ankles down to her toes and mildly reduced sense of vibration distally in her toes with normal position sense. Motor examination was normal. DTR was normal in the upper and lower extremities. Serum protein electrophoresis and immunofixation were normal. EMG/NCS was negative for large fiber peripheral neuropathy. Unfortunately, the patient did not undergo a skin biopsy for evaluation of small fiber neuropathy due to insurance-related issues. She failed multiple neuropathic pain medications, including gabapentin, pregabalin, and duloxetine, due to various side effects (dizziness, tiredness, and drowsiness), and reported partial improvement with venlafaxine 37.5 mg.

Teriflunomide therapy was discontinued, and lower extremity pain and paresthesia stabilized without progression.

Case 3

A 47-year-old woman with an eight-year history of RRMS presented with neuropathy in her hands and feet after six months of teriflunomide treatment. She reported numbness as well as tingling and sharp pain. Before teriflunomide therapy, she had failed trials of DMTs due to various side effects, including injection site reactions with glatiramer acetate, worsening depression and flu-like symptoms with Rebif, and severe leukopenia with fingolimod. 

Neurological examination showed features of small fiber neuropathy with reduced sensation to pinprick in a stocking-and-glove pattern with a mildly reduced sense of vibration distally in her toes and a normal position sense. Motor examination was normal. DTR was normal in both the upper and lower extremities. Serum protein electrophoresis and immunofixation were normal. EMG/NCS was negative for large fiber peripheral neuropathy. A skin biopsy revealed significantly reduced ENFD in a sample taken from the lateral right thigh, consistent with small fiber neuropathy, and low-normal ENFD from the right calf (Table 2). The patient partially responded to the gabapentin 600 mg three times/day.

**Table 2:** Epidermal nerve fiber density test results for patient 3 (nerve fiber per millimeter)

Specimen	Patient value	Abnormal	Low normal
Right thigh	6.81	<8.3	8.3–9.1
Right calf	5.32	<4.8	4.8–5.5

Teriflunomide therapy was discontinued, and lower extremity pain and paresthesia stabilized without progression.

In all three patients, repeat neuroimaging (including MRI of the brain, cervical, and thoracic spine with and without contrast with demyelinating protocol) revealed chronic demyelinating disease without new or active demyelinating lesions.

## Discussion

We discuss the cases of three patients with clinical features suggestive of painful distal symmetric small fiber peripheral neuropathy after teriflunomide treatment. Since the medication’s integration into the market in 2012 until 2022, the FDA’s online database of adverse effects received 427 reports of peripheral neuropathy associated with teriflunomide [7]. Additionally, the product’s summary of characteristics discloses an incidence rate of peripheral neuropathy of 1.4% and 1.9% in patients receiving 7 mg and 14 mg of teriflunomide, respectively [8]. Yet, there is scarce data regarding these patients in the literature. In contrast to teriflunomide, there is a growing literature documenting peripheral neuropathy associated with the mother drug, leflunomide (Tables 3, 4) [3,6,9-15].

**Table 3:** Literature review of peripheral neuropathy cases associated with teriflunomide use RRMS: relapsing-remitting multiple sclerosis; EMG: electromyography; ENFD: epidermal nerve fiber density

Study	Patient age (years), sex (M/F)	Geographic location	Medication prescribed	Indication of treatment, duration of disease	Maintenance dosage	Time to onset	Comorbidities	Medications	Clinical presentation	Electrodiagnostic testing	Outcome	Mean follow-up time
Comi et al., 2016[6]. Analysis of 4 clinical trials (30 patients)	N/A	Italy	Teriflunomide	N/A	7 or 14 mg/day	N/A	N/A	N/A	Variable	N/A	Drug cessation in 8 patients	N/A
Borrelli et al., 2021 [14]. Cross-sectional analysis of 9 patients	Mean age: 41, 66% M	Belgium	Teriflunomide	RRMS, secondary-progressive multiple sclerosis (SPMS), mean: 8 years	N/A	N/A	N/A	Previous treatment with glatiramer acetate, dimethyl fumarate beta-interferon in 6 patients	4 patients (44%) presented with symptoms compatible with a peripheral neuropathy score ≥3	3 patients (33%) had very mild and isolated abnormalities	Teriflunomide was administered for a mean duration of 34 months	N/A
O’Connor et al., 2016 [15]. Nine-year follow-up of TEMSO study (14 patients)	18–55 years, N/A	116 countries	Teriflunomide	Multiple sclerosis	7 or 14 mg/day	N/A	N/A	N/A	N/A	N/A	8 ongoing events, 3 resolved with sequelae (mean duration: 267 days), 4 resolved without sequelae (mean duration: 429 days)	9 years
Current study (patient 1)	61, F	USA	Teriflunomide	RRMS, 8 years	14 mg/day	4 years	History of diverticulitis, anxiety	Previous treatment with glatiramer acetate, interferon beta-1a, dimethyl fumarate	Burning sensation in the legs from the calves to the feet; tingling and sharp shooting pains in feet	EMG was negative for large fiber neuropathy. Biopsy showed reduced ENFD	Failed to improve upon drug cessation	6 months
Current study (patient 2)	54, F	USA	Teriflunomide	RRMS, 3 years	14 mg/day	1 year	History of chronic migraines, anxiety/depression	Previous treatment with glatiramer acetate and dimethyl fumarate	Sharp stabbing pain, tingling in the legs from the calves down to the feet	EMG was negative for large fiber neuropathy	Failed to improve upon drug cessation	2 years
Current study (patient 3)	47, F	USA	Teriflunomide	RRMS, 8 years	14 mg/day	<6 months	History of chronic migraines, anxiety/depression	Previous treatment with glatiramer acetate and fingolimod	Numbness, tingling, sharp pains in the hands and feet	EMG was negative for large fiber neuropathy. Biopsy showed reduced ENFD	Failed to improve upon drug cessation	2 years

**Table 4:** Literature review of peripheral neuropathy cases associated with leflunomide use NSAIDs: nonsteroidal anti-inflammatory drugs; EMG: electromyography

Study	Patient age (years), sex (M/F)	Geographic location	Medication prescribed	Indication of treatment, duration of disease	Maintenance dosage	Time to onset	Comorbidities	Medications	Clinical presentation	Electrodiagnostic testing	Outcome	Mean follow-up time
Bonnel and Graham, 2004 [3]. Case series of 80 case reports	Mean age 62, 61% F	61% from the US, the rest from elsewhere	Leflunomide	Rheumatoid arthritis, psoriatic arthritis, connective tissue disease, dermato-myositis, polyarthritis	10 to 20 mg/day	Mean: 6 months, range: 3 days to 3 years	History of diabetes mellitus, neuropathy, hypothyroidism, lung cancer, spinal stenosis in some patients	Previous treatment with cisplatin, sulfasalazine, chloroquine, isoniazid, and ticlopidine in some patients. Concomitant treatment with NSAIDs, warfarin, losartan, and tamoxifen in 14 patients	Peripheral numbness, tingling or burning, severe pain, cold sensation in distal extremities, or extremity weakness	Distal axonal, sensory, or sensorimotor polyneuropathy in 37 patients. A biopsy in 1 patient showed axonal loss without vasculitis	18% were hospitalized, 21% improved or recovered upon drug cessation, 8 patients failed to improve after cholestyramine	N/A
Antonio-Valdiviezo et al., 2010 [9]. Case report	48, F	Mexico	Leflunomide	Rheumatoid arthritis, 3 years	20 mg/day	4 months	Diabetes mellitus type 2	Previous treatment with anti-inflammatory drugs, methotrexate, sulfasalazine. Concomitant treatment with chloroquine, prednisone	Dysesthesia in the left cubital area, paresthesia in the right knee, hyporeflexia, and decreased muscle strength in the left forearm, right hand, face, and right leg	Severe segmental demyelinating polyneuropathy, retrograde axonal degeneration on EMG and electroneurophysiological studies	Failed to improve after 10 months of drug cessation and 1 month of cholestyramine	2.5 years
Hill, 2004 [10]. Case report	36, F	Australia	Leflunomide	Psoriatic arthritis	10 mg/day, which was increased to 20 mg/day	3 months	N/A	Previous treatment with sulphasalazine and methotrexate. Current treatment with rofecoxib and omeprazole	Numbness and paresthesia in feet, hands, mid-forearm, and mid-calf. Normal power with reduced pinprick, temperature, and light touch	N/A	Complete resolution of symptoms upon drug cessation and cholestyramine treatment	2 weeks
Kho and Kermode, 2007 [11]. Case report	60, F	Australia	Leflunomide	Polyarthralgia/myalgia syndrome	20 mg/day	4 years	N/A	Previous treatment with methotrexate	Numbness over the halluces, the plantar surfaces, and all toes of both feet, electric shock-like feeling, decreased knee jerk and absent ankle jerk responses bilaterally, decreased pinprick and light touch	Mild sensorimotor axonal neuropathy on EMG	Failed to improve upon drug cessation	12 months
Kho and Kermode, 2007 [11]. Case report	65, M	Australia	Leflunomide	Rheumatoid arthritis, 5 years	20 mg/day	Within weeks	N/A	Previous treatment with prednisolone, methotrexate	Loss of sensation in the feet, absent ankle jerk responses, loss of light touch sensation in the upper and lower limbs, decreased pinprick sensation, absent vibration sense in the knee	Peripheral sensorimotor axonal neuropathy in the upper and lower limbs on EMG. A sural nerve biopsy showed severe active and chronic axonopathy with secondary Wallerian degeneration	Failed to improve upon drug cessation	18 months
Gabelle et al., 2005 [12]. Case report	61, F	France	Leflunomide	Rheumatoid arthritis, 3 years	20 mg/day	5 months	N/A	Previous treatment with prednisone and methotrexate. Concomitant treatment with paracetamol, enalapril, hydrochlorothiazide, venlafaxine, alprazolam, omeprazole	Hypoesthesia bilaterally in the lower limb, severe paraparesis, bilateral amyotrophy, absent osteotendinous reflexes in the lower limbs	Asymmetric axonal neuropathy	Improvement in walking and regression of amyotrophy upon drug cessation	8 months
Gabelle et al., 2005 [12]. Case report	70, F	France	Leflunomide	Rheumatoid arthritis, N/A	20 mg/day	5 months	History of asthma, angina, hypercholesterolemia	Previous treatment with corticosteroids. Concomitant treatment with molsidomine, aspirin, calcium, vitamin D3, omeprazole, simvastatin, vitamin P	Paresthesia and burns of the feet, hands, and face, significant allodynia on contact. No motor deficits	Absent sensory potential in the musculocutaneous nerves and right sural nerve; decreased amplitude in the left sural nerve	Complete resolution of dysesthesias of the hands; persistence of minimal symptoms in the feet upon drug cessation	5 months
Carulli and Davies, 2002 [13]. Case report	76, M	UK	Leflunomide	Rheumatoid arthritis, 1.5 years	10 mg/day	2 weeks	History of chronic emphysema, pulmonary fibrosis	Previous treatment with azathioprine. Concomitant treatment with prednisolone, tramadol, Didronel PMO, indoramin, celecoxib	Polymyalgic symptoms, with a stocking distribution up to the malleoli	Sensorimotor damage and axonal degeneration on EMG	Improved upon drug cessation	3 months
Carulli and Davies, 2002 [13]. Case report	69, F	UK	Leflunomide	Rheumatoid arthritis, 10 years	10 mg/day	3 months	N/A	Previous treatment with gold salts, methotrexate. Concomitant treatment with prednisolone, lansoprazole, simvastatin, losartan, amiodarone	Numbness in the fingertips and feet bilaterally	Sensorimotor peripheral neuropathy on EMG	Improved upon drug cessation	3 months

The exact mechanism by which leflunomide may cause neuropathy remains unknown. Bonnel and Graham (2004) attributed the patients’ neuropathy to the accumulation of toxic metabolites generated due to leflunomide’s inhibitory effect on uridine production [3]. By blocking uridine diphosphate (UDP)-glucuronosyltransferases (UGT), leflunomide impedes the conjugation of endogenous compounds, xenobiotics, and toxic substances with glucuronic acid and thereby obstructs their excretion [16]. On the other hand, Carulli and Davies (2002) suggested that the drug may provoke vasculitic neuropathy, which was confirmed by a sural nerve biopsy [13]. Bharadwaj and Haroon (2004) reinforced this hypothesis by obtaining nerve biopsies from three leflunomide-treated patients presenting with peripheral neuropathy [17]. Epineural perivascular inflammation and prominent neovascularization established the presence of axonal neuropathy and vasculitis in them.

Several factors favor teriflunomide as the culprit in our patients. There was a temporal relationship between the initiation of teriflunomide treatment and the onset of the neurological complaints. Moreover, symptoms stabilized following drug cessation. Additionally, EMG results ruled out large fiber neuropathy, and biopsies performed in patients 1 and 2 revealed a reduction in ENFD, establishing a diagnosis of small fiber neuropathy. Laboratory investigations showed no evidence of primary or secondary vasculitis in any of the patients. Other possible neuropathy causes - including metabolic or immune disorders, vitamin deficiency, and concomitant use of neurotoxic drugs - have been ruled out in all three cases (Table 5).

**Table 5:** Laboratory data ANA: antinuclear antibody; C-ANCA: antineutrophil cytoplasmic autoantibody, cytoplasmic; Citr Pep: citrullinated peptide; RF: rheumatoid factor; P-ANCA: perinuclear antineutrophil cytoplasmic antibodies; HDL: high-density lipoprotein; LDL: low-density lipoprotein; TSH: thyroid-stimulating hormone

Variables	Reference values	Case 1	Case 2	Case 3
ANA	Negative	Positive	Positive	Positive
ANA pattern		Homogenous	Homogenous	Homogenous
ANA titer 1	<1:80 titer	1:320	1:320	1:320
C-ANCA	<1:20 titer	<1:20	<1:20	<1:20
Cyclic Citr Pep IgG	<7 IU/mL	1.8	1.2	4.2
DNA Ab (ds)	Negative	Negative	Negative	Negative
RF by nephelometry	<14 IU/mL	<10	<10	<10
P-ANCA	<1:20 titer	<1:20	<1:20	<1:20
C3 complement	90–230 mg/dL	158	130	99
C4 complement	10–51 mg/dL	32	2.0	42
RNP antibody	<20 units	2	5	3
SM antibody	<20 units	2	6	4
SS A/Ro antibody	<30 units	4	4	14
SS B/La antibody	<20 units	2	5	13
Cholesterol	120–200 mg/dL	246, high	__	182
Triglyceride	40–200 mg/dL	218, high	__	58
HDL cholesterol	>40 mg/dL	59	__	66
LDL cholesterol	70–130 mg/dL	143, high	__	124
Vitamin B12	180 - 810 pg/mL	598	854	637
Glycated hemoglobin	<5.7%	5.6	5.2	5.0
Glucose, fasting	60–99 mg/dL	103, high	95	97
free light chains ratio	0.26–1.65	1.61	__	__
Kappa light chains	3.3–19.4 mg/L	14.5	__	__
Lambda light chains	5.7–26.3 mg/L	9.0	__	__
IgG, serum	700–1,600 mg/dL	642, low	1101	824
IgA, serum	70–400 mg/dL	75	360	__
IgM, serum	40–230 mg/dL	59	109	__
Protein, total, serum	6.58–8.51 g/dL	6.5, low	7.4	5.8, low
Albumin	3.73–5.65 g/dL	4.37	4.3	4.9
Beta globulins	0.69–1.29 g/dL	0.67, low	3.5	2.1
M protein	<0.0 g/dl	<0.1 g/dl (IgG lambda)	0	0
TSH	0.45–5.33 uIU/mL	1.44	2.58	3.11
Cardiolipin AB IgA	<12 APL	__	<9.0	<9.0
Cardiolipin AB IgG	<15 GPL	__	<9.0	<9.0
Cardiolipin AB IgM	<12.5 MPL	__	<9.0	12.0
HIV 4th generation antigen/antibody		__	Non-reactive	Non-reactive

The patients had no history of diabetes or hypertension. Autoimmune workup was only positive for antinuclear antibodies (ANA) (homogeneous pattern 1:320), a non-specific finding since all other autoimmune labs were normal. HIV testing was not recommended for patient 1 as the patient had no risk for HIV contraction and yielded negative results for patients 2 and 3. Lastly, patient 1 had chronic metabolic syndrome long before teriflunomide use, and her neuropathy began after the drug intake. It is unclear if the MGUS diagnosis had any link with teriflunomide exposure, as the patient did not have any testing done for MGUS before the diagnosis of small fiber neuropathy was established.

## Conclusions

Teriflunomide-induced neuropathy is not only underreported in literature but also underrecognized in clinical practice. In cases of suspected neuropathy following teriflunomide exposure, early diagnosis and prompt treatment cessation may result in better clinical outcomes. Failure to improve upon drug cessation could imply irreversible damage. Clinicians should thus discuss this rare side effect with their patients to ensure timely management of small fiber neuropathy and prevent its progression to large fiber neuropathy. Failure to do so will cause patients to resort to polypharmacy for the management of neuropathic pain, which has detrimental consequences on their health, such as weight gain, sedation, drowsiness, and fatigue.

## References

[1] Misra UK, Kalita J, Nair PP (2008). Diagnostic approach to peripheral neuropathy. Ann Indian Acad Neurol.

[2] Zaottini F, Picasso R, Pistoia F (2022). High-resolution ultrasound of peripheral neuropathies in rheumatological patients: an overview of clinical applications and imaging findings. Front Med (Lausanne).

[3] Bonnel RA, Graham DJ (2004). Peripheral neuropathy in patients treated with leflunomide. Clin Pharmacol Ther.

[4] Bayas A, Mäurer M (2015). Teriflunomide for the treatment of relapsing-remitting multiple sclerosis: patient preference and adherence. Patient Prefer Adherence.

[5] Bar-Or A, Pachner A, Menguy-Vacheron F, Kaplan J, Wiendl H (2014). Teriflunomide and its mechanism of action in multiple sclerosis. Drugs.

[6] Comi G, Freedman MS, Kappos L (2016). Pooled safety and tolerability data from four placebo-controlled teriflunomide studies and extensions. Mult Scler Relat Disord.

[7] (2023). FDA Adverse Event Reporting System (FAERS) Public Dashboard. https://www.fda.gov/drugs/questions-and-answers-fdas-adverse-event-reporting-system-faers/fda-adverse-event-reporting-system-faers-public-dashboard.

[8] Sanofi Sanofi (2023). Aubagio summary of product characteristics. https://products.sanofi.us/aubagio/aubagio.pdf.

[9] Antonio-Valdiviezo A, Peña-Santos G, Martínez-Torres J (2010). Peripheral neuropathy caused by leflunomide. A case reported with a brief review (Article in Spanish). Rev Med Inst Mex Seguro Soc.

[10] Hill CL (2004). Leflunomide-induced peripheral neuropathy: rapid resolution with cholestyramine wash-out. Rheumatology (Oxford).

[11] Kho LK, Kermode AG (2007). Leflunomide-induced peripheral neuropathy. J Clin Neurosci.

[12] Gabelle A, Antoine JC, Hillaire-Buys D, Coudeyre E, Camu W (2005). Leflunomide-related severe axonal neuropathy (Article in French). Rev Neurol (Paris).

[13] Carulli MT, Davies UM (2002). Peripheral neuropathy: an unwanted effect of leflunomide?. Rheumatology (Oxford).

[14] Borrelli S, Pereira Lima J, Dachy B (2023). Borrelli S, Lima JP, Dachy B: peripheral neuropathy as potential side effect of teriflunomide. https://www.charcot-ms.org/files/05_ECF2021_Poster_CL_Borrelli_S.pdf.

[15] O'Connor P, Comi G, Freedman MS (2016). Long-term safety and efficacy of teriflunomide: nine-year follow-up of the randomized TEMSO study. Neurology.

[16] Sanchez-Dominguez CN, Gallardo-Blanco HL, Salinas-Santander MA, Ortiz-Lopez R (2018). Uridine 5'-diphospho-glucronosyltrasferase: its role in pharmacogenomics and human disease. Exp Ther Med.

[17] Bharadwaj A, Haroon N (2004). Peripheral neuropathy in patients on leflunomide. Rheumatology (Oxford).

